# Thrombin Upregulates PAI-1 and Mesothelial–Mesenchymal Transition Through PAR-1 and Contributes to Tuberculous Pleural Fibrosis

**DOI:** 10.3390/ijms20205076

**Published:** 2019-10-13

**Authors:** Cheng-Ying Hsieh, Joen-Rong Sheu, Chih-Hao Yang, Wei-Lin Chen, Jie-Heng Tsai, Chi-Li Chung

**Affiliations:** 1 Department of Pharmacology, School of Medicine, College of Medicine, Taipei Medical University, Taipei 110, Taiwan; hsiehcy@tmu.edu.tw (C.-Y.H.); sheujr@tmu.edu.tw (J.-R.S.); chyang@tmu.edu.tw (C.-H.Y.); 2 Graduate Institute of Medical Sciences, College of Medicine, Taipei Medical University, Taipei 110, Taiwan; 3 Department of Nursing, Mackay Junior College of Medicine, Nursing, and Management, Taipei 112, Taiwan; s519@mail.mkc.edu.tw; 4 School of Nutrition and Health Sciences, College of Public Health and Nutrition, Taipei Medical University, Taipei 110, Taiwan; a19851102@hotmail.com; 5 Division of Pulmonary Medicine, Department of Internal Medicine, Taipei Medical University Hospital, Taipei 110, Taiwan; 6 Division of Pulmonary Medicine, Department of Internal Medicine, School of Medicine and School of Respiratory Therapy, College of Medicine, Taipei Medical University, Taipei 110, Taiwan

**Keywords:** mesothelial–mesenchymal transition, plasminogen activator inhibitor-1, pleural fibrosis, pleural mesothelial cell, residual pleura thickening, thrombin, tuberculous pleural effusion, tuberculous pleural fibrosis

## Abstract

Thrombin is an essential procoagulant and profibrotic mediator. However, its implication in tuberculous pleural effusion (TBPE) remains unknown. The effusion thrombin and plasminogen activator inhibitor-1 (PAI-1) levels were measured among transudative pleural effusion (TPE, *n* = 22) and TBPE (*n* = 24) patients. Pleural fibrosis, identified as radiological residual pleural thickening (RPT) and shadowing, was measured at 12-month follow-up. Moreover, in vivo and in vitro effects of thrombin on PAI-1 expression and mesothelial–mesenchymal transition (MMT) were assessed. We demonstrated the effusion thrombin levels were significantly higher in TBPE than TPE, especially greater in TBPE patients with RPT > 10mm than those without, and correlated positively with PAI-1 and pleural fibrosis area. In carbon black/bleomycin-treated mice, knockdown of protease-activated receptor-1 (PAR-1) markedly downregulated α-smooth muscle actin (α-SMA) and fibronectin, and attenuated pleural fibrosis. In pleural mesothelial cells (PMCs), thrombin concentration-dependently increased PAI-1, α-SMA, and collagen I expression. Specifically, *Mycobacterium tuberculosis* H37Ra (MTBRa) induced thrombin production by PMCs via upregulating tissue factor and prothrombin, and PAR-1 silencing considerably abrogated MTBRa−stimulated PAI-1 expression and MMT. Consistently, prothrombin/PAR-1 expression was evident in the pleural mesothelium of TBPE patients. Conclusively, thrombin upregulates PAI-1 and MMT and may contribute to tuberculous pleural fibrosis. Thrombin/PAR-1 inhibition may confer potential therapy for pleural fibrosis.

## 1. Introduction

Tuberculosis (TB) remains a major global public health issue [1]. Tuberculous pleural effusion (TBPE) is the most common extrapulmonary TB and often complicates with residual pleural fibrosis that results in pulmonary restriction and dyspnea [2]. TBPE, caused by tuberculous pleural injury, is enriched with inflammatory cells, various cytokines, and coagulation factors [3]. There is increasing evidence demonstrating the correlation between these indicators and the severity of inflammation and subsequent pleural fibrosis [4,5]. However, the pathogenesis and optimal management of tuberculous pleural fibrosis remain to be elucidated. 

Pleural fibrosis arises from pleural injury that activates two key profibrotic mechanisms including formation of fibrin neomatrix and proliferation of myofibroblasts on fibrin strands with production of extracellular matrix (ECM) in the pleural space [2,6]. The pleural space is lined by a monolayer of pleural mesothelial cells (PMCs) that serve as the first-line defense against invading pathogens and modulate inflammation and tissue repair [7]. In response to injury, PMCs elaborate tissue factor (TF) to activate coagulation and produce plasminogen activator inhibitor-1 (PAI-1) to suppress fibrinolysis, resulting in pleural fibrin deposition [7,8]. Moreover, recent in vitro and in vivo studies showed that, upon pleural injury, PMCs undergo mesothelial–mesenchymal transition (MMT) into myofibroblasts and secrete excess ECM to develop pleural fibrosis [9,10]. All these findings indicate that PAI-1 overexpression and MMT in PMCs are essential in pleural fibrogenesis. Intriguingly, a recent animal study reported that PAI-1 deficiency paradoxically promotes pleural injury, MMT and organization, which highlight the key role of PAI-1 in tissue repair and merit further investigations [11]. 

Thrombin is not only an essential procoagulant but also a powerful profibrotic mediator and has been implicated in inflammation and fibrosis of various organs including liver, lung, heart and kidney [12,13]. In the context of pleural injury, induction of coagulation cascade generates a thrombin burst to convert soluble fibrinogen into insoluble fibrin [6]. Moreover, thrombin can induce TF and reduce TF pathway inhibitor expression in PMCs to augment fibrin deposition in the pleural space [14,15]. Notably, thrombin exerts profibrotic effects via activation of the G-protein coupled, protease-activated receptors (PARs) [16], and a recent study demonstrated that thrombin induces mesenchymal transformation and ECM production in PMCs through PAR-1-mediated signaling, indicating the important role of PAR-1 in modulation of pleural fibrosis [9]. However, regarding PMC-dependent fibrinolysis, there existed inconsistent results of thrombin regulation on PAI-1 expression in PMCs [11,17]. All the aforementioned preclinical data suggest that thrombin may be substantially involved in the pathogenesis of pleural inflammation and fibrosis. Moreover, there is growing in vivo evidence of targeting thrombin/PAR-1 axis as a promising treatment for pulmonary fibrosis [18,19]. Nevertheless, to the best of our knowledge, the profibrotic role and clinical significance of thrombin and PAR-1 signaling in TBPE have never been investigated. This study aimed to examine the in vitro and in vivo effects of thrombin/PAR-1 signaling on PAI-1 expression and MMT in human PMCs and the clinical implications in TBPE.

## 2. Results

### 2.1. Thrombin Levels Between Transudative Pleural Effusion and Tuberculous Pleural Effusion

Consecutive 46 patients with TPE (*n* = 22) and TBPE (*n* = 24) were enrolled (Table 1), including 29 men and 13 women with an age range from 20 to 91 years. All patients with TPE were diagnosed with congestive heart failure, and to explore the profibrotic role of thrombin in TBPE, the TBPE patients were categorized into residual pleural thickening (RPT) ≤ 10 mm (*n* = 14) and RPT > 10 mm (*n* = 10) groups, based on the chest radiograph at the end of 12-month follow-up. All patients finished 12 months of follow-up from September 2014 through August 2016. 

**Figure 1:**
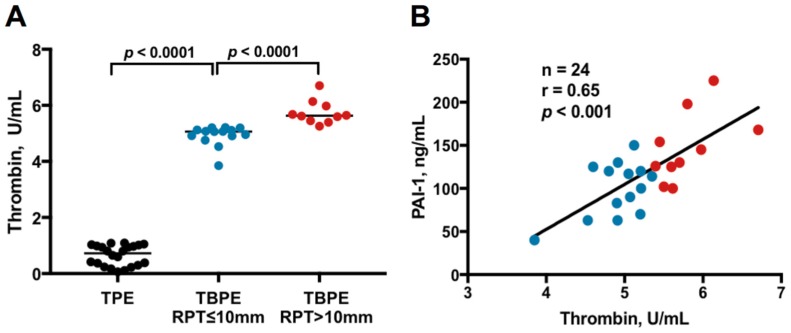
Thrombin levels among TPE, TBPE with RPT ≤ 10 mm and TBPE with RPT > 10 mm groups and the correlation between thrombin and PAI-1 in TBPE (**A**) Pleural effusion thrombin levels were significantly higher in TBPE than in TPE and were markedly higher in TBPE patients with RPT > 10 mm than in those with RPT ≤ 10 mm. (**B**) Pleural effusion thrombin levels were positively correlated with those of PAI-1 in TBPE. TPE, transudative pleural effusion (*n* = 22); TBPE, tuberculous pleural effusion (total *n* = 24); RPT, residual pleural thickening; PAI-1, plasminogen activator inhibitor-1. Blue dot, TBPE with RPT ≤ 10 mm (*n* = 14); Red dot, TBPE with RPT > 10 mm (*n* = 10).

As shown in Table 1 and Figure 1A, the median levels of effusion thrombin were significantly higher in both TBPE groups than in TPE group (RPT ≤ 10 mm group vs. TPE group, *p* < 0.0001; RPT > 10 mm group vs. TPE group, *p* < 0.0001), which implies a pathogenic role of thrombin in TBPE. Furthermore, the effusion thrombin level was remarkably higher in TBPE patients with RPT >10 mm (5.7 pg/mL, range 5.4–6.7 pg/mL) than those with RPT ≤ 10 mm (5.0 pg/mL, range 3.9–5.4 pg/mL) (*p* < 0.0001).

### 2.2. Cytokines and Fibrinolytic Factors between TBPE Patients with Residual Pleural Thickening (RPT) ≤ 10 mm and RPT > 10 mm

Accordingly, we further compared the pleural fluid characteristics, proinflammatory cytokines and fibrinolytic factors between the two TBPE groups (Table 1). The pleural fluid parameters demonstrated that RPT > 10 mm group had significantly lower levels of effusion pH and higher level of adenosine deaminase (ADA) than did RPT ≤ 10 mm group, while there was no considerable difference in pleural fluid values of glucose, lactate dehydrogenase (LDH), and leukocyte count between two groups. Moreover, besides thrombin, the effusion levels of plasminogen activator inhibitor (PAI)-1, tumor necrosis factor (TNF)-α and interleukin (IL)-1β were significantly higher in RPT > 10 mm group than in RPT ≤ 10 mm group. Additionally, the former had greater initial effusion chest radiograph (CXR) score and lower forced vital capacity at 12 months than the latter. In parallel with our previous report [20], the higher initial effusion CXR score may represent greater pleural inflammation and fluid exudation in patients with RPT > 10 mm. These findings suggest that the increased inflammation, decreased fibrinolysis and especially the elevated thrombin are associated with development of pleural fibrosis in TBPE. 

### 2.3. Correlation Between Thrombin and Inflammatory Parameters, Fibrinolytic Factors and Cytokines in TBPE

Accordingly, to explore the link between thrombin and inflammation, fibrinolysis and fibrosis in TBPE, we examined the relationship between thrombin and inflammatory parameters, fibrinolytic factors and other cytokines among TBPE patients (Figure 1B, Table 2). The results demonstrated that the effusion levels of thrombin were positively correlated with those of PAI-1 (*r* = 0.65, *p* < 0.0001) and tended to have negative correlation with pH value (*r* = −0.46, *p* = 0.051). However, there was no significant correlation between thrombin and glucose, LDH, tissue-type plasminogen activator (tPA), TNF-α and IL-1β, respectively. The current data suggest that thrombin is associated with increased inflammation and reduced fibrinolysis in TBPE.

### 2.4. Multivariate Logistic Regression Analysis

To further ascertain the importance of thrombin in fibrosis of TBPE, multivariate logistic regression analysis was used to identify the factors associated with RPT > 10 mm in TBPE after 12-month follow-up (Table 3). Variables of significance in univariate analysis were included for analysis and thrombin and PAI-1 were treated separately because they were mutually correlated. This demonstrated that only higher effusion thrombin and PAI-1 levels were independent predictors for RPT > 10 mm in TBPE.

### 2.5. Optimal Sensitivity, Specificity, and Cutoff Value of Variables to Predict RPT > 10 mm

**Figure 2:**
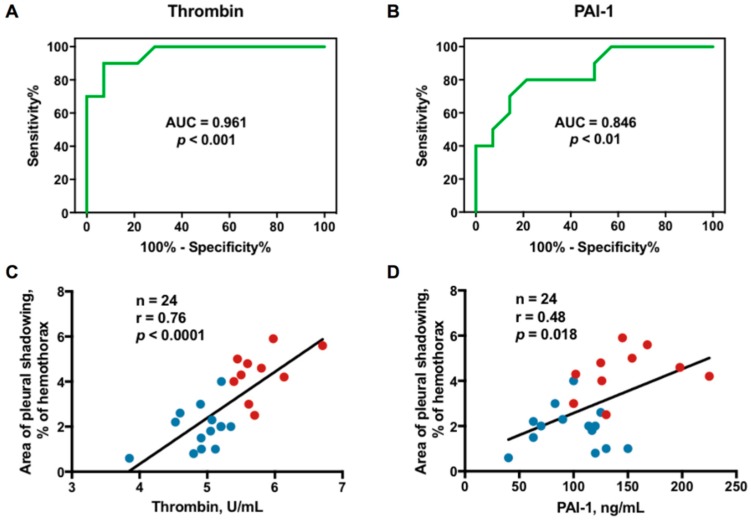
Relationship between effusion thrombin or PAI-1 and residual pleural fibrosis in TBPE: (**A**) receiver operating characteristic (ROC) curve for effusion thrombin level to predict RPT > 10 mm in TBPE; (**B**) ROC curve for effusion PAI-1 level to predict RPT > 10 mm in TBPE; (**C**) Correlation between effusion thrombin level and residual pleural shadowing CXR score in patients with TBPE; (**D**) Correlation between effusion PAI-1 level and residual pleural shadowing CXR score in patients with TBPE. AUC, area under the ROC curve; PAI-1, plasminogen activator inhibitor-1; RPT, residual pleural thickening; TBPE, tuberculous pleural effusion. Blue dot, TBPE with RPT ≤ 10 mm (*n* = 14); Red dot, TBPE with RPT > 10 mm (*n* = 10).

Furthermore, the receiver operating characteristic (ROC) curves showed that the effusion thrombin at the cutoff level >5.4 pg/mL had highest sensitivity and specificity for predicting RPT > 10 mm in TBPE patients (area under the ROC curve = 0.961, 95% confidence interval (CI) = 0.892–1.029; sensitivity 90%, 95% CI = 55.5–97.5%; specificity 92.9%, 95% CI = 66.1–99.8%) (Figure 2A), followed by PAI-1 at the cutoff level >125.5 pg/mL (area under the ROC curve = 0.846, 95% CI = 0.689–1.004; sensitivity 70%, 95% CI = 34.8–93.3%; specificity 85.7%, 95% CI = 57.2–98.2%) (Figure 2B).

### 2.6. Correlation Between Effusion Thrombin or Plasminogen Activator Inhibitor-1 (PAI-1) and Residual Pleural Fibrosis

Given the predictive value of effusion thrombin and PAI-1, the correlation between thrombin or PAI-1 and residual pleural fibrosis were analyzed. As shown in Figure 2C,D, the effusion levels of both, especially thrombin, were positively correlated with the CXR score of pleural shadowing at 12 months, suggesting that thrombin is essentially implicated in fibrogenesis of TBPE that warrants further experiments to verify.

### 2.7. Protease-Activated Receptor (PAR)-1 Silencing Attenuates Pleural Fibrosis in Vivo

**Figure 3:**
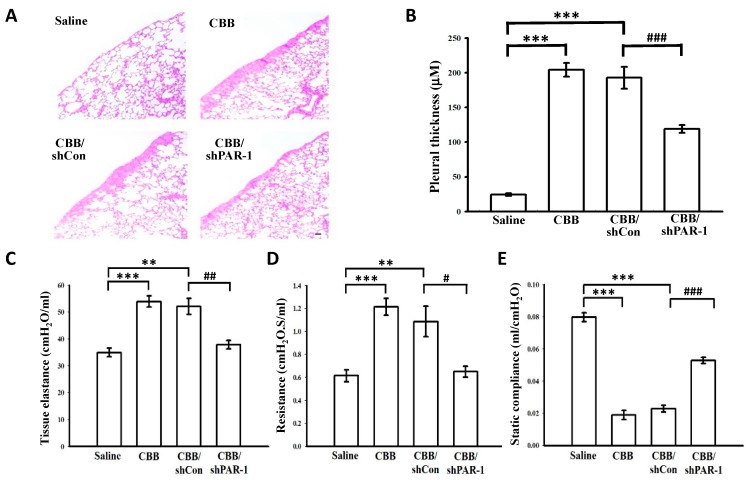
Effects of PAR-1 silencing on pleural thickening and lung function in CBB-induced pleural fibrosis mice model. C57/B6 mice were treated with saline, CBB, CBB plus control shRNA (shCon) or CBB plus PAR-1 shRNA (shPAR-1) for 14 days. (**A**) The lung tissue sections were harvested and stained with hematoxylin and eosin to determine pleural thickening. The scale bar indicates 50 μm. (**B**) Pleural thickness from the stained lung sections was measured and collected from 10 fields per slide (*n* = 3 representative sections per group). (**C**–**E**) The assessment of lung function including lung elastance (**C**), resistance (**D**), and compliance (**E**) was also performed. Data are presented as the means ± SEM (*n* = 3). *** *p* < 0.001 compared with the saline control group; ^#^ *p* < 0.05, ^##^ *p* < 0.01, and ^###^ *p* < 0.001, compared with the CBB/shCon group. Carbon black/bleomycin (CBB); PAR-1, protease-activated receptor-1; shRNA, short hairpin RNA.

The thrombin receptor PAR-1 but not PAR-3 or -4 has been reported to be highly expressed in human pleuritic lung sections, and has been known to mediate thrombin signaling in pleural mesothelial cells [9]. To explore the profibrotic role of thrombin, we thus silenced PAR-1 by intratracheal administration of PAR-1 short hairpin RNA (shRNA) to determine the effect of thrombin on pleural thickening and lung function in carbon black/bleomycin (CBB) pleural fibrosis mice model. As shown in Figure 3A,B, the CBB treatment significantly induced pleural thickening compared to the saline control group. In addition, CBB-treated mice revealed substantially increased tissue elastance (saline control group: 35.02 ± 1.62 cmH_2_O/mL vs. CBB/shCon group: 53.95 ± 2.09 cmH_2_O/mL; *p* < 0.01) and resistance (saline control group: 0.62 ± 0.05 cmH_2_O.S/mL vs. CBB/shCon group: 1.21 ± 0.07 cmH_2_O.S/mL; *p* < 0.01) (Figure 3C,D), whereas static compliance was diminished (saline control group: 0.080 ± 0.005 mL/cmH_2_O vs. CBB/shCon group: 0.019 ± 0.003 mL/cmH_2_O; *p* < 0.001) (Figure 3E). Conversely, the pleural thickening and lung function impairment were markedly reversed by administration of PAR-1 shRNA in CBB-treated mice (Figure 3A–E). These findings suggest that the thrombin receptor PAR-1 essentially mediates pleural injury and resultant fibrosis in CBB pleural fibrosis model.

### 2.8. Thrombin Induces Mesothelial–Mesenchymal Transition in Vivo and in Vitro Through PAR-1, PI3K/AKT and NF-κB Signalings 

**Figure 4:**
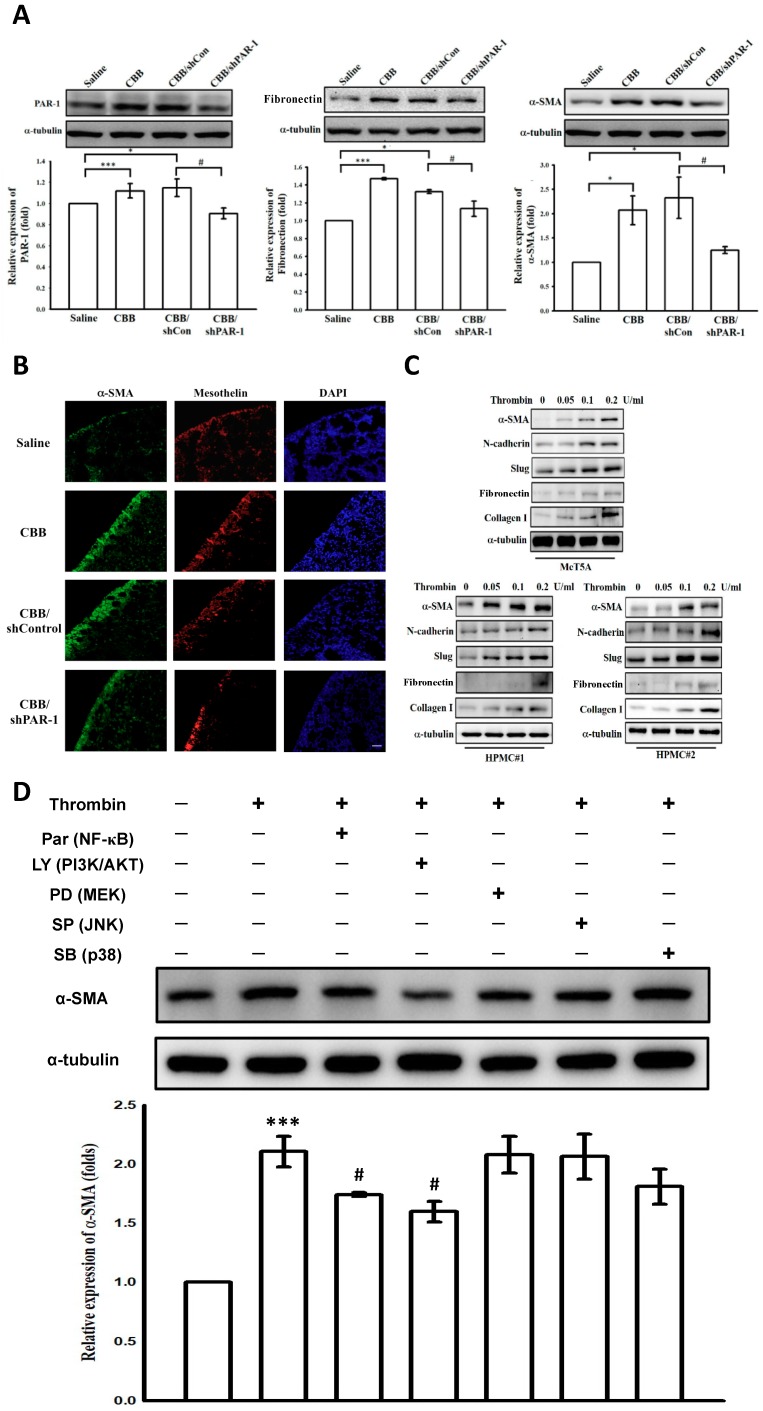
Thrombin induced MMT in CBB-injured mice and human PMCs possibly through PAR-1, PI3K/AKT and NF-B signaling pathways. C57/B6 mice were treated with saline, CBB, CBB plus control shRNA (shCon) or CBB plus PAR-1 shRNA (shPAR-1) for 14 days. (**A**) The homogenates of lung tissue were subjected to Western blotting assay to determine the expression of α-SMA, fibronectin, and collagen I. * *p* < 0.5, *** *p* < 0.001 compared with the saline control group; ^#^ *p* < 0.05 compared with the CBB/shCon group. (**B**) Immunostaining of α-SMA (green) or mesothelin (red) was performed in the lung tissue sections. Cells were also co-stained with 4′, 6-diamidino-2-phenylindole (DAPI) to visualize the nuclei (blue). Scale bar, 50 μm. (**C**) The expression of MMT markers including α-SMA, N-cadherin, slug, fibronectin, and collagen I were evaluated in MeT-5A cells and primary human pleural mesothelial cells (HPMCs) treated with thrombin for 24 h by Western blotting assay. (**D**) MeT-5A cells were pretreated with different signaling pathway inhibitors including parthenolide (Par), LY294002 (LY), PD98059 (PD), SP600125 (SP), and SB203580 (SB) followed by the stimulation with thrombin (0.2 U/mL) for 24 h, and the expression of α-SMA was determined by Western blotting assay. Figures are representative examples of three independent experiments. *** *p* < 0.001 compared with the control group; ^#^ *p* < 0.05 compared with the thrombin group. Carbon black/bleomycin (CBB); PAR-1, protease-activated receptor-1; shRNA, short hairpin RNA; PMC, pleural mesothelial cell; MMT, mesothelial–mesenchymal transition; PI3K, phosphatidylinositol 3-kinases; AKT, protein kinase B; NF-κB, nuclear factor-κB; MEK, mitogen-activated protein kinase kinase; JNK, c-Jun *N*-terminal kinases; p38, p38 mitogen-activated protein kinase; α-SMA, α-smooth muscle actin.

To clarify the mechanism whereby thrombin mediates pleural fibrosis, we further investigated the effects of thrombin on MMT in CBB-injured mice and in human PMCs. As shown in Figure 4A, CBB treatment apparently upregulated PAR-1 and induced the expression of fibronectin and α-smooth muscle actin (α-SMA) in the lung section homogenate of mice. Contrarily, administration of PAR-1 shRNA substantially suppressed the expression of these MMT markers. Furthermore, the immunostaining of α-SMA revealed the occurrence of MMT in the pleural mesothelium of CBB-treated mice, and silencing of PAR-1 significantly attenuated α-SMA expression in the pleural mesothelium (Figure 4B). On the other hand, the expression of MMT markers including α-SMA, N-cadherin, Slug, fibronectin and collagen I was also evaluated in vitro. As shown in Figure 4C, thrombin concentration-dependently stimulated expression of theses MMT markers in both MeT-5A cells and primary PMCs. In addition, pretreatment with neither a MEK inhibitor (PD98059; 20 μM), a JNK inhibitor (SP600125; 10 μM), nor a p38 MAPK inhibitor (SB203580; 10 μM) reduced the expression of α-SMA in thrombin (0.2 U/mL)-treated MeT-5A cells. In contrast, a PI3K/AKT inhibitor (LY294002; 20 μM) and a NF-κB inhibitor (Parthenolide; 10 μM) significantly abolished α-SMA expression (Figure 4D). Collectively, these findings indicate that thrombin induced MMT in vivo and in vitro and resulted in pleural fibrosis possibly through activation of PAR-1, PI3K/AKT and NF-κB signalings.

### 2.9. Thrombin Induces PAI-1 Expression in Human Pleural Mesothelial Cells Through PAR-1/JNK Signaling 

**Figure 5:**
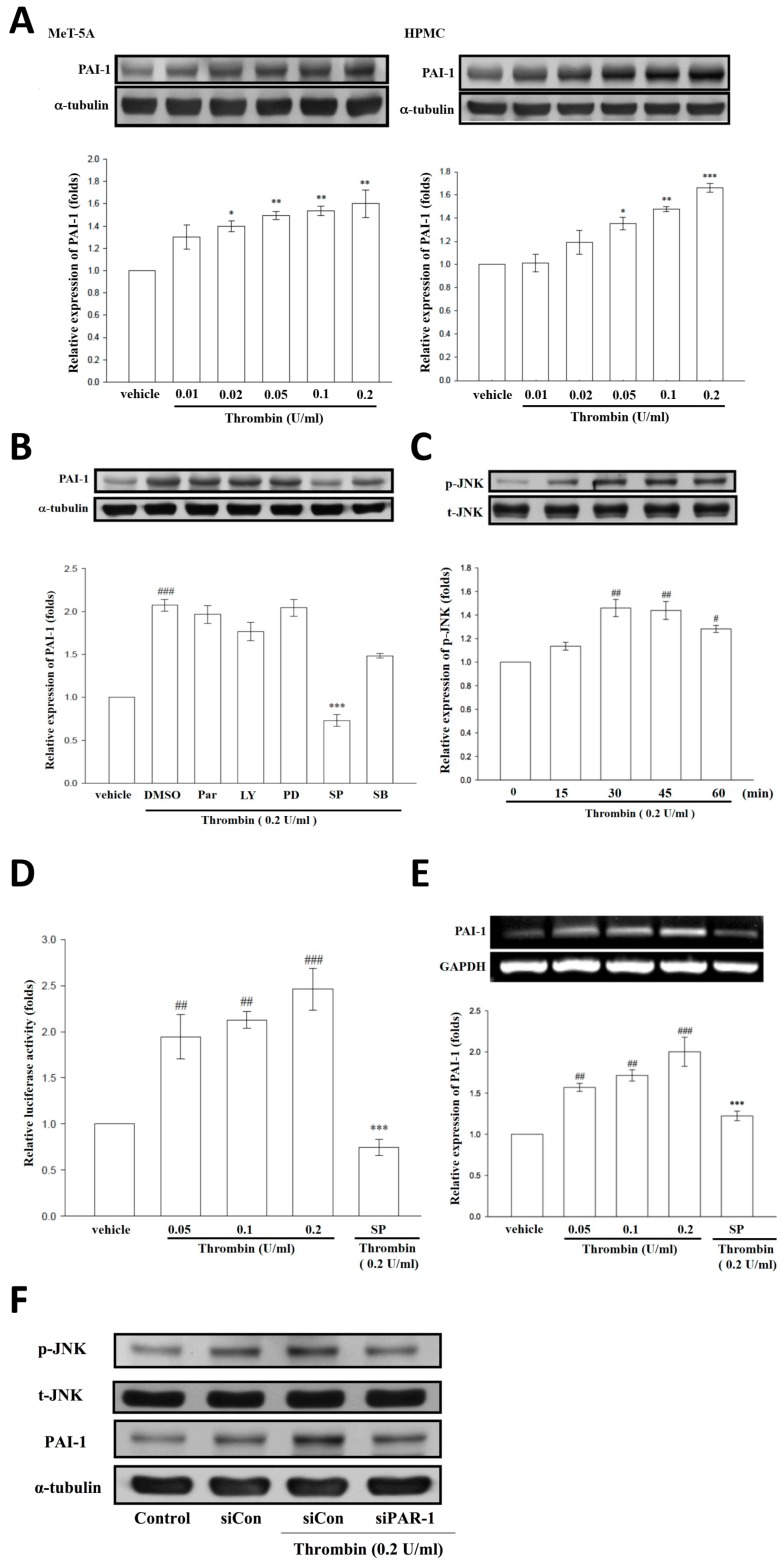
Thrombin induces PAI-1 expression in human PMCs through PAR-1/JNK signaling. (**A**) Treatment with thrombin (0.01, 0.02, 0.05, 0.1, and 0.2 U/mL) for 24 h triggered PAI-1 expression in both MeT-5A cells and primary human pleural mesothelial cells (HPMCs). * *p* < 0.05, ** *p* < 0.01, *** *p* < 0.001 compared with vehicle group; (**B**) PAI-1 expression was determined by Western blotting assay in thrombin-stimulated MeT-5A cells pretreated with DMSO (dimethyl sulfoxide) or different signaling pathway inhibitors including parthenolide (Par, 10 μM), LY294002 (LY, 20 μM), PD98059 (PD, 20 μM), SP600125 (SP, 10 μM), and SB203580 (SB, 10 μM). ^###^ *p* < 0.001 compared with vehicle group, *** *p* < 0.001 compared with DMSO group; (**C**) The JNK phosphorylation was evaluated in MeT-5A cells treated with thrombin (0.2 U/mL) for the indicated times by Western blotting assay. ^#^ *p* < 0.05, ^##^ *p* < 0.01, compared with resting group; (**D**–**E**) The reporter activity and mRNA level of PAI-I were assessed as described in “Methods and Materials” in thrombin (0.05, 0.1, 0.2 U/mL)-stimulated MeT-5A cells pretreated with or without SP600125 (10 μM). ^##^ *p* < 0.01, ^###^ *p* < 0.001 compared with vehicle group, *** *p* < 0.001 compared with the thrombin (0.2 U/mL) group (**F**) The JNK phosphorylation and PAI-1 expression were measured by Western blotting assay in MeT-5A cells pretreated with control siRNA (siCon) or PAR-1 siRNA (siPAR-1) followed by thrombin (0.2 U/mL) stimulation for 24 h. Data are presented as the means ± SEM (*n* = 3). PAI-1, plasminogen activator inhibitor-1; PMC, pleural mesothelial cell; PAR-1, protease-activated receptor-1; JNK, c-Jun *N*-terminal kinases; GAPDH, glyceraldehyde 3-phosphate dehydrogenase; siRNA, small interfering RNA.

As shown in Figure 1B, the levels of thrombin were positively correlated with PAI-1 in TBPE, we thus further determined the molecular link between thrombin and PAI-1 in human PMCs. Treatment with thrombin (0.01–0.2 U/mL) significantly triggered PAI-1 expression in both MeT-5A cells and primary human PMCs in a concentration-dependent manner (Figure 5A). Moreover, thrombin-induced PAI-1 expression was significantly diminished by pretreatment with a JNK inhibitor (SP600125) but not by a NF-κB inhibitor (Parthenolide), a PI3K inhibitor (LY294002), a MEK inhibitor (PD98059), nor a p38 MAPK inhibitor (SB203580) in MeT-5A cells (Figure 5B). Consistently, thrombin time-dependently stimulated JNK phosphorylation (Figure 5C), and pretreatment with SP600125 substantially repressed the promoter activity and mRNA level of PAI-1 in thrombin-treated MeT-5A cells (Figure 5D,E). Furthermore, PAR-1 silencing effectively abrogated thrombin-mediated JNK phosphorylation and PAI-1 expression (Figure 5F). These data signify that thrombin upregulates PAI-1 expression in human PMCs through PAR-1/JNK signaling and thereby disrupts fibrinolysis in the process of pleural injury.

### 2.10. Heat-Killed Mycobacterium Tuberculosis H37Ra Increased Thrombin Production in Human PMCs 

**Figure 6:**
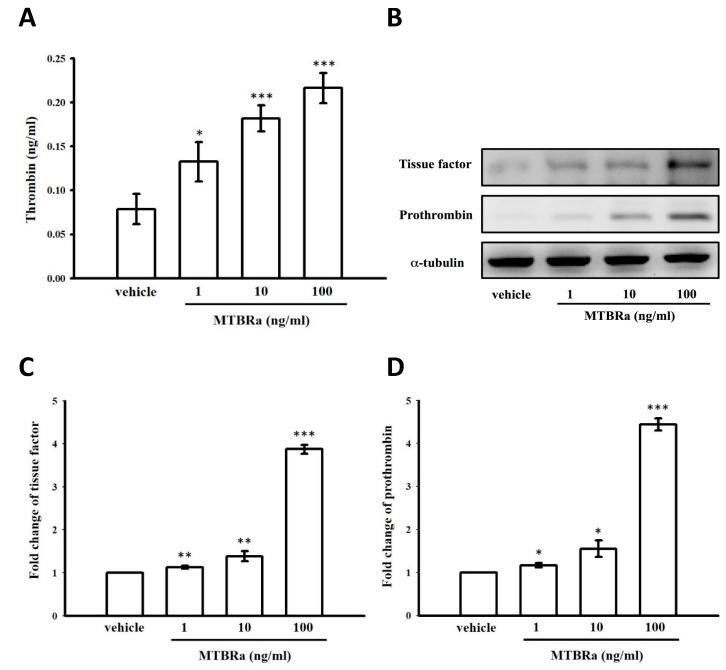

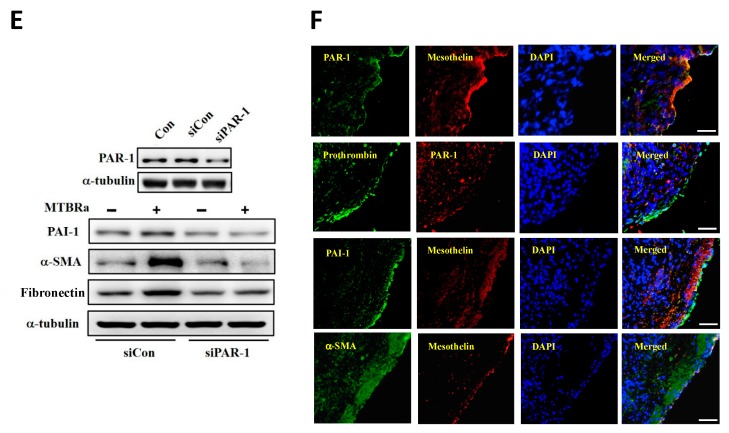
MTBRa induces expression of tissue factor and prothrombin to increase production of thrombin that upregulates PAI-1 expression and MMT through PAR-1 in human PMCs and associates with expression of PAR-1, prothrombin, PAI-1 and α-SMA in pleural mesothelium of patients with TBPE. (**A**) The thrombin level in the conditioned medium of MTBRa-treated MeT-5A cells was measured by ELISA. (**B**) The expression of tissue factor and prothrombin in MTBRa-treated MeT-5A cells was assessed by Western blotting assay. (**C**, **D**) The mRNA level of tissue factor and prothrombin in MTBRa-treated MeT-5A cells was assessed by semi-quantitative reverse transcriptase PCR. (**E**) The expression of PAI-1, α-SMA and fibronectin was determined by Western blotting assay in MeT-5A cells pretreated with control siRNA (siCon) or PAR-1 siRNA (siPAR-1) followed by the stimulation of MTBRa (10 ng/mL) for 24 h. (**F**) Immunofluorescence staining of mesothelin (red), PAR-1 (green or red), prothrombin (green), PAI-1 (green), or α-SMA (green) was performed on the paraffin-embedded pleural mesothelium from patients with TBPE. Cells were also co-stained with DAPI to visualize nuclei (blue). Scale bar, 100 μm. Data are presented as the means ± SEM (*n* = 3). * *p* < 0.05, ** *p* < 0.01, *** *p* < 0.001 compared with vehicle group. MTBRa, *Mycobacterium tuberculosis* H37Ra; PAI-1, plasminogen activator inhibitor-1; MMT, mesothelial–mesenchymal transition; PAR-1, protease-activated receptor-1; PMC, pleural mesothelial cell; siRNA, small interfering RNA; α-SMA, α-smooth muscle actin; DAPI, 4′, 6-diamidino-2- phenylindole.

Given that thrombin can induce PAI-1 expression and MMT (Figure 3, Figure 4 and Figure 5) and that the thrombin level is markedly elevated and correlates with pleural fibrosis in TBPE (Figure 1A and Figure 2C), we further investigated the generation mechanism of thrombin in TBPE using *Mycobacterium tuberculosis* H37Ra (MTBRa) as the stimulant of tuberculous pleural injury. As shown in Figure 6A, MTBRa (1–100 ng/mL) concentration-dependently induced thrombin production in the conditioned medium of MeT-5A cells.

### 2.11. MTBRa Induced mRNA and Protein Expression of Tissue Factor and Prothrombin in Human PMCs

The procoagulant response in pleural injury occurs largely via overexpression of TF and activation of extrinsic coagulation pathway that subsequently convert prothrombin into thrombin [13]. In order to elucidate the cause of raised production of thrombin in TBPE, we examined the effect of MTBRa on TF and prothrombin expression in PMCs, and found that MTBRa significantly increased the mRNA and protein expression of TF and prothrombin in a concentration-dependent manner (Figure 6B–D), which may further trigger the coagulation cascade and raised the level of thrombin.

### 2.12. MTBRa Upregulates PAI-1, α-SMA and Fibronectin Expression Through PAR-1 in Human PMCs 

Moreover, MTBRa significantly stimulated PAI-1, α-SMA, and fibronectin expression in MeT-5A cells, and knockdown of PAR-1 apparently abolished all the effects (Figure 6E). All these findings (Figure 6A–E) indicate that upon MTB infection, human PMCs are activated to produce a considerable amount of thrombin that, through PAR-1 mediation, subsequently impairs fibrinolysis and induces MMT and ECM production in TBPE.

### 2.13. Expression of PAR-1, Prothrombin, PAI-1, and α-SMA in the Pleural Mesothelium of Patients with TBPE

Furthermore, the immunofluorescence staining of mesothelin, prothrombin, PAR-1, PAI-1 or α-SMA was performed on the pleural mesothelium specimen biopsied from TBPE patients (Figure 6F), and demonstrated proliferation of PMCs (mesothelin positive cells) that exhibited substantial dual expression of PAR-1^+^/mesothelin^+^, prothrombin^+^/PAR-1^+^, PAI-1^+^/mesothelin^+^ and α-SMA^+^/mesothelin^+^, which further indicates the important implication of thrombin, PAI-1 and MMT in the pathogenesis of TBPE.

**Figure 7:**
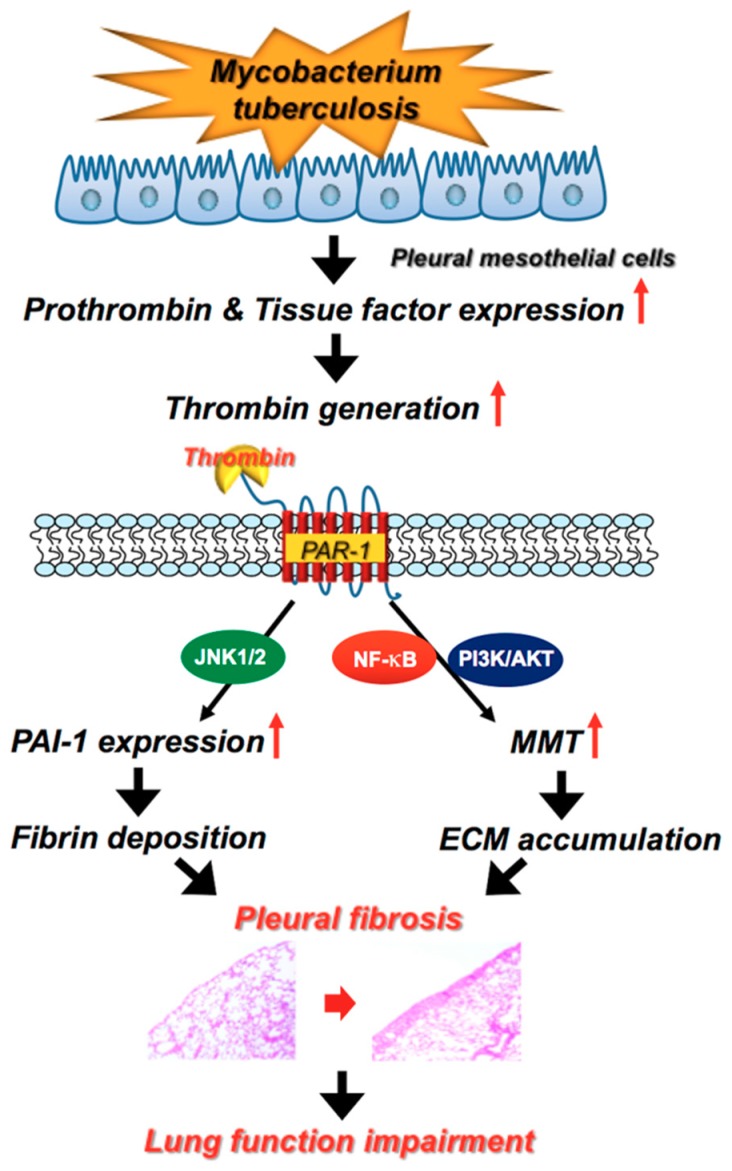
Schematic diagram of the activated signalings of PAI-1 expression and MMT in human pleural mesothelial cells upon *Mycobacterium tuberculosis* infection and thrombin stimulation. *Mycobacterium tuberculosis* triggers tissue factor and prothrombin expression to generate thrombin that activates PAR-1 to elicit PAI-1 overexpression and fibrin deposition and to induce MMT and ECM accumulation possibly via JNK and PI3K/AKT and NF-κB signaling pathways, respectively, and ultimately gives rise to pleural fibrosis in TBPE (see text for further explanation). PAI-1, plasminogen activator inhibitor-1; MMT, mesothelial–mesenchymal transition; PAR-1, protease-activated receptor-1; JNK, c-Jun *N*-terminal kinases; NF-κB, nuclear factor-κB; PI3K, phosphatidylinositol 3-kinases; AKT, protein kinase B; ECM, extracellular matrix; TBPE, tuberculous pleural effusion.

Collectively, all these results demonstrated that *M. tuberculosis* infection triggers pleural injury, upregulates TF and prothrombin, and thus increases thrombin production in the pleural space. Subsequently, thrombin activates PMCs through PAR-1 to elicit PAI-1 overexpression and fibrin deposition and to induce MMT and ECM elaboration, and ultimately gives rise to pleural fibrosis in TBPE (Figure 7). JNK, PI3K/AKT and NF-κB signaling pathways may be involved in the cellular process. More works are required to completely characterize the intracellular signalings in the thrombin-stimulated PMCs.

## 3. Discussion

The current study demonstrated that the effusion levels of thrombin were significantly higher in TBPE than TPE, and especially greater in TBPE patients with RPT > 10 mm than those with RPT ≤ 10 mm. Moreover, thrombin correlated positively with PAI-1 and both had positive correlation with pleural fibrosis area and were independent predictors for RPT > 10 mm in TBPE. The in vivo experiment showed that knockdown of PAR-1 markedly diminished CCB-induced α-SMA and fibronectin expression in the pleural mesothelium, and significantly attenuated pleural fibrosis and reversed lung function impairment. In human PMCs, thrombin concentration-dependently increased the expression of MMT markers including α-SMA, N-cadherin, Slug, fibronectin and collagen I through PI3/AKT and NF-κB signalings. Furthermore, thrombin upregulated PAI-1 mRNA and protein expression via activation of JNK pathway, while pretreatment with PAR-1 siRNA effectively repressed JNK phosphorylation and PAI-1 synthesis in PMCs. Specifically, MTBRa concentration-dependently induced thrombin production by PMCs via upregulating TF and prothrombin, and PAR-1 silencing considerably abrogated MTBRa−stimulated expression of PAI-1, α-SMA and fibronectin. Consistently, PAR-1, prothrombin, PAI-1 and α-SMA were notably expressed concomitantly in the pleural mesothelium of TBPE patients. To our knowledge, this is the first study to signify the clinical significance and profibrotic implication of thrombin/PAR-1-mediated dual upregulation of PAI-1 and MMT in TBPE. 

Thrombin is the central protease of the coagulation cascade. Following tissue injury, thrombin exerts multiple procoagulant effects including conversion of fibrinogen to fibrin, activation of factors V, VIII, IX, XIII, upregulation of TF and downregulation of TF pathway inhibitor [14,15,21]. Thrombin also inhibits fibrinolysis through activation of thrombin-activable fibrinolysis inhibitor and induction of PAI-1 expression [21]. Moreover, thrombin can induce mesenchymal transformation of pulmonary fibroblasts, vascular endothelial cells and alveolar epithelial cells into myofibroblasts [22,23,24]. Accordingly, thrombin plays a key role in the interplay among coagulation, inflammation and fibrosis [21], and the thrombin level or activity is significantly increased in bronchoalveolar fluids of patients with lung injury, systemic sclerosis and idiopathic pulmonary fibrosis [12,13,25], indicating the involvement of thrombin in altered tissue remodeling. However, the role of thrombin in TBPE has rarely been investigated. A previous study revealed that thrombin-antithrombin III complex is raised and associated with severity of inflammation in TBPE [4], implying the participation of thrombin in TB pleural infection. Furthermore, the current study first demonstrated that the thrombin level was markedly elevated in TBPE compared to TPE and that MTBRa stimulated thrombin production in human PMCs, signifying the role of thrombin in the pathogenesis of TBPE.

Consequently, to verify the clinical implication of thrombin in pleural fibrosis, we assessed the residual pleural shadowing of TBPE patients and found that, among all the measured pleural fluid variables, both the effusion thrombin and PAI-1 were profoundly higher in patients with RPT > 10 mm than those with RPT ≤ 10 mm. In parallel, the correlation analysis showed that thrombin had strong positive correlation with PAI-1 in TBPE. However, although the effusion levels of pH, ADA, TNF-α and IL-1β were also substantially raised in RPT > 10 mm group compared to RPT ≤ 10 mm group, there existed only weak but not significant correlation between thrombin and these inflammatory parameters. In addition, both thrombin and PAI-1 were independent predictors for RPT > 10 mm and had positive correlation with the area of residual pleural shadowing of TBPE, suggesting that both thrombin and PAI-1 are essential for tuberculous pleural fibrosis. Intriguingly, compared to PAI-1, thrombin was a better predictor for RPT > 10 mm and had stronger correlation with residual pleural opacity. As previous studies have demonstrated the upregulation effect of thrombin on PAI-1 [11,26], the current results imply that, besides increasing PAI-1 expression to impair fibrinolysis and enhance fibrin neomatrix, thrombin may possess other profibrotic functions, such as induction of aberrant cellular response to injury, to promote pleural fibrosis in TBPE [22,23,24].

Jeffers et al. indicated that PAR-1, but not PAR-3 and -4, was expressed markedly higher in human pleuritic lung sections than in normal human pleura. In addition, they detected the distinct PAR-1 expression in the pleural mesothelium in CBB-induced pleural injury mouse model, indicating that PAR-1 is the main receptor mediating thrombin-involved pleural injury [15]. Accordingly, to clarify the implication of thrombin in pleural fibrogenesis, we further explored the role of PAR-1 signaling in vivo and in vitro. The results revealed that administration of PAR-1 shRNA substantially attenuated the pleural thickening and lung function impairment in CBB-induced pleural fibrosis model. On the other hand, in parallel with the previous in vitro report showing that thrombin induced MMT [9], we again demonstrated that thrombin concentration-dependently upregulated MMT markers in PMCs, and blockade of PI3K/AKT and NF-κB significantly attenuated thrombin–stimulated α-SMA expression. Moreover, we conducted in vivo experiments to further disclose the increased expression of α-SMA and fibronectin in CBB-mediated pleural injury, and the reduction of pleural MMT and ECM production by PAR-1 silencing. These data highlight the key role of thrombin/PAR-1 signaling in driving MMT in pleural fibrogenesis. 

The regulation of thrombin on PMC-dependent fibrinolysis remains controversial. A former report showed that thrombin inhibited fibrinolysis not through induction of PAI-1 but via cleavage and inactivation of single chain urokinase-type plasminogen activator [17]. In contrast, a recent study revealed that thrombin significantly increases PAI-1 synthesis in PMCs and that PAI-1 overexpression augmented intrapleural fibrin deposition in CBB-injured mouse model [11]. In parallel with the latter report [11], we demonstrated a strong positive correlation between the levels of thrombin and PAI-1 and their link with pleural fibrosis in TBPE, and further verified that thrombin concentration-dependently induced PAI-1 mRNA and protein expression in PMCs through activation of JNK signaling pathway. Moreover, PAR-1 silencing effectively mitigated JNK phosphorylation and PAI-1 production. These results may feature the importance of thrombin/PAR-1 axis in upregulation of PAI-1 expression and fibrin deposition in pleural injury. 

Given that thrombin mediates dual upregulation of PAI-1 expression and MMT in PMCs, the mechanism by which thrombin is greatly elevated and associated with pleural fibrosis in TBPE remains to be elucidated. The current study revealed that MTBRa stimulated PMCs to elaborate tissue factor and prothrombin and may thereby initiate tissue factor-induced coagulation and ultimately convert prothrombin into thrombin in the pleural space, which may explicate the elevation of thrombin in TBPE. Moreover, we demonstrated the prominent expression of PAR-1, prothrombin, PAI-1 and α-SMA in the pleural mesothelium of patients with TBPE and that MTBRa upregulated PAI-1, α-SMA and fibronectin via PAR-1 signaling in human PMCs. Collectively, this study first showed that expression of prothrombin was increased in human PMCs upon MTB infection and indicated that thrombin/PAR-1 signaling mediates PAI-1 overexpression, MMT and ECM synthesis and may thus contribute to pleural fibrosis in TBPE. 

As for the therapeutic implication of thrombin for pleural fibrosis, previous reports and our study have demonstrated that direct thrombin inhibition that ablates PAR-1 signaling efficiently attenuated thrombin-mediated alveolar EMT, pleural MMT and PAI-1 expression in vitro [9,24,27], and mitigated bleomycin-induced lung and pleural fibrosis in vivo [28,29]. Moreover, the preliminary result of the ongoing human trial showed that the clinically available direct thrombin inhibitor, Dabigatran etexilate, is safe and effectively reduces α-SMA expression in lung and skin fibroblasts in systemic sclerosis-associated interstitial lung disease patients [30], indicating the therapeutic potential of PAR-1 antagonist for pulmonary fibrosis. Accordingly, more in vivo and clinical studies to inhibit thrombin/PAR-1 signaling are warranted for developing novel agents for treatment of pleural fibrosis.

## 4. Materials and Methods 

### 4.1. Materials

Fetal calf serum (FCS), Roswell park memorial institute (RPMI) medium, medium 199 (M199), L-glutamine penicillin/streptomycin, trypsin (0.25%), and PAR-1 and control siRNA were purchased from Thermo Fisher Scientific Inc. (Waltham, MA, USA). Thrombin, bleomycin, and carbon black were acquired from Sigma-Aldrich Corporation (St. Louis, MO, USA). Nuclear factor-κB (NF-κB) inhibitor: parthenolide, phosphatidylinositol 3-kinases/protein kinase B (PI3K/AKT) inhibitor: LY294002, mitogen-activated protein kinase kinase (MEK) inhibitor: PD98059, c-Jun *N*-terminal kinases (JNK) inhibitor: SP600125, and p38 mitogen-activated protein kinase (MAPK) inhibitor: SB203580 were obtained from Calbiochem (San Diego, CA, USA). DAPI-Fluoromount-G was purchased from Southern Biotech. (Birmingham, AL, USA). Heat-killed *Mycobacterium tuberculosis* H37Ra (MTBRa) (Difco Lab, Detroit, MI, USA) was dissolved in phosphate-buffered saline (PBS) and stored at −80 °C until used. 

The antibodies to α-SMA and N-cadherin were purchased from Cell Signaling Technology (Beverly, MA), and those to collagen I, fibronectin, mesothelin were obtained from Santa Cruz Biotechnology Inc. (Dallas, TX, USA). The antibody to PAI-1 was from BD Biosciences (San Jose, CA, USA). The antibody to prothrombin was purchased from Abcam (Cambridge, UK). The primary antibody to α-tubulin, goat anti-rabbit secondary IgG (Alexa Fluor 488) and goat anti-mouse secondary IgG (Alexa Fluor 594) were from Thermo Fisher Scientific (Waltham, MA, USA). The horseradish peroxidase (HRP)-conjugated donkey anti-rabbit IgG, and sheep anti-mouse IgG were purchased from Amersham (Buckinghamshire, UK). The Hybond^®^-P polyvinylidene difluoride (PVDF) membranes and Western blotting detection reagent of enhanced chemiluminescence were purchased from GE Healthcare Life Sciences (Waukesha, WI, USA).

### 4.2. Patient Selection

Consecutive patients with pleural effusion admitted to Taipei Medical University Hospital were eligible and included if TBPE or TPE was diagnosed. TBPE was diagnosed by the demonstration of granulomatous pleuritis on closed pleura biopsy specimens with or without the presence of acid-fast bacilli. Ethics approval CRC-05–11-01 was obtained from the Institutional Review Board of Taipei Medical University Hospital (Taipei, Taiwan). All patients presented written informed consent prior to entering the study. Exclusion criteria included history of invasive pleural procedures, recent severe trauma, hemorrhage or stroke; bleeding diathesis or anticoagulant therapy.

### 4.3. Thoracentesis and Pleural Fluid Analysis 

Immediately or within 24 h after hospitalization, 50 mL of pleural fluid was aspirated with the guidance of chest ultrasonography. Pleural fluid analyses, ADA, microbiological studies were performed routinely. Standard anti-TB medications for six months were administered once TBPE was diagnosed.

### 4.4. Chest Radiographs and Pulmonary Function 

Posterior–anterior CXRs were taken on admission and every two months during the follow-up period up to 12 months. CXRs were saved as a digital image in the picture archiving and communication system (PACS) and the area occupied by the pleural opacity and the hemithorax were measured using an image-processing program provided by PACS. Each CXR was read and scored by two independent radiologists who were blinded to any clinical information to determine (a) lateral pleural thickening: the largest linear width of pleural opacity and (b) CXR score of the size of pleural effusion or thickening: the estimated overall percentage of pleural shadowing in the hemithorax [5]. Clinically significant RPT was defined as a lateral pleural thickening of >10 mm shown on CXR, which was further evidenced by chest computed tomography, at the end of 12-month follow-up [31]. Pulmonary function tests with spirometry were performed at 12 months following the initiation of treatment.

### 4.5. Measurement of Cytokines and Fibrinolytic Factors 

The commercially available enzyme-linked immunosorbent assay (ELISA) kits were used to measure the levels in pleural effusions or in PMC conditioned medium of thrombin (AssayPro LLC., St. Charles, MO, USA), TNF-α, IL-1β, PAI-1, and tPA (R & D System; Minneapolis, MN, USA) as previously described [20].

### 4.6. Carbon Black/Bleomycin Pleural Fibrosis Mouse Model 

The mouse model of CBB-induced pleural fibrosis was performed as previously described with some modifications [10]. Male C57BL/6 mice (8-week-old) purchased from BioLASCO (Taipei, Taiwan) received intratracheal instillation with 100 μL of CBB mixture (0.1 mg carbon black/0.07 unit bleomycin dissolved in normal saline) to induce pleural fibrosis [10]. After 14 days, mice were transferred to the FlexiVent (SCIREQ) system for forced oscillations measurements. A maneuver of snapshot perturbation was imposed to measure tissue resistance, static compliance, and elastance of the whole respiratory system (airways, lung, and chest wall). Briefly, the mice were anaesthetized and underwent tracheotomy. The trachea was inserted with the previously calibrated cannula (1.2 cm, 18 gauges) and ventilation was provided with a tidal volume of 10 mL/kg, a rate of 150 breaths/min, and a positive end-expiratory pressure of 3 cm H_2_O. Then lung function parameters were measured by fitting pressure and volume data to the single compartment model and analyzed with flexiWare 7 Software [32]. The mice were then sacrificed and pleural thickening were then evaluated in the lung tissue sections. The Institutional Animal Care and Use Committee of Taipei Medical University approved all animal experiments and care procedures.

### 4.7. Cell Cultivation of Pleural Mesothelial Cells

Primary human PMCs and MeT-5A human pleural mesothelial cell line (ATCC^®^ CRL-9444™; ATCC, Manassas, VA, USA) were both utilized in this study. The primary human PMCs were harvested from pleural fluids of patients with congestive heart failure as previously described [5]. Primary human PMCs and MeT-5A cells were incubated in RPMI and M199 medium, respectively, in a humidified atmosphere of 5% CO_2_ at 37 °C. The mediums were both supplemented with 10% FCS, penicillin G (100 units/mL), streptomycin (100 mg/mL), and L-glutamine (2 mM).

### 4.8. Protease-Activated Receptor-1 Silencing

To verify the role of thrombin and its major receptor PAR-1 in pleural fibrosis, PAR-1 silencing was implemented in vivo and in vitro. C57BL/6 mice received intratracheal injection with control shRNA or PAR-1 shRNA before administration of CBB. PAR-1 (clone TRCN0000220247) and control (clone TRCN0000072243) shRNA in the pLKO.1-puro vector were purchased from National RNAi Core Facility, Academia Sinica, Taiwan. The used titer of this vector was 5×10^8^ transducing unit (TU)/mL, determined as previously described [33]. MeT-5A cells were seeded in a 60 mm dish. The medium was replaced with serum-free Opti-MEM (Gibco BRL, Grand Island, NY, USA) when the cells reached confluence, then the cells were transiently transfected with control small interfering RNA (siRNA) or PAR-1 siRNA (Thermo Fisher Scientific, Inc., USA) for a final concentration of 100 nM per culture using Lipofectamine™3000 reagent in accordance with the instructions of the manufacturer’s protocol (Thermo Fisher Scientific, Inc., USA).

### 4.9. Western Blotting Assay

Protein lysates were separated through sodium dodecyl sulfate-polyacrylamide gel electrophoresis and transferred to a PVDF membrane by using a semidry transfer unit (Bio-Rad; Hercules, CA). The membranes were then blocked with TBST (10 mM Tris-base, 100 mM NaCl, and 0.01% Tween 20) containing 5% skimmed milk for 1 h at room temperature, and then probed with various specific primary antibodies for 2 h. The membranes were washed 5 min for three times and then incubated with the HRP-linked secondary antibody for 1 h. Immunoreactive bands were exposed by an enhanced chemiluminescence system. The ratios of the semiquantitative results were analyzed by a videodensitometer and Bio-profil Biolight software (version V2000.01; Vilber Lourmat, Marne-la-Vallée, France).

### 4.10. PAI-1 Luciferase Activity Assay

MeT-5A cells were cotransfected with the PAI-1 and Renilla reporter plasmid using the TurboFect transfection reagent (Thermo Fisher Scientific; Waltham, MA, USA). After a 24 h period of incubation, cells were lysed and luciferase activity was measured as described previously [34]. 

### 4.11. Reverse Transcription–Polymerase Chain Reaction

Total RNA of MeT-5A cells was extracted by the TRIsure^®^ reagent (Bioline; London, UK), and RNA (1 μg) was subjected to cDNA synthesis (Super Script On-Step RT-PCR system, Thermo Fisher Scientific; Waltham, MA, USA). PCR products were resolved on agarose gels and were stained by ethidium bromide. Specific primer sequences (sense/antisense) were designed as follows: PAI-1: 5′-TGCTGGTGAATGCCCTCTACT-3′/5′-CGGTCATTCCCAGGTTCTCTA-3′; Prothrombin: 5′-TGGAATCCTACATCGACGGG-3′/5′-TCGGTGAAGTTCTTGTCCCA-3′; Tissue factor: 5′-AGTGATTCCCTCCCGAACAG-3′/5′-AGTTCTCCTTCCAGCTCTGC-3′; GAPDH: 5′-GCCGCCTGGTCACCAGGGCTG-3′/5′-ATGGACTGTGGTCATGAGCCC-3′.

### 4.12. Immunofluorescence Staining Assay

To determine the expression of PAR-1, prothrombin, PAI-1 and α-SMA in the pleural mesothelium, the biopsy specimen of parietal pleura tissue from patients with TBPE were embedded in paraffin then de-waxed by xylene and alcohols. The slices were immunostained with primary antibody to mesothelin, PAR-1, prothrombin, PAI-1 or α-SMA, and then probed by Alexa Fluor 488 and Alexa Fluor 594-conjugated secondary antibody, respectively. The stained slices were counterstained and mounted by DAPI-Fluoromount-G mounting buffer (Southern Biotech, Birmingham, AL, USA), and then subjected to the TCS SP5 confocal spectral microscope imaging system (Leica, Wetzlar, Germany).

### 4.13. Statistical Analysis

Quantitative data are presented as median (range) or mean ± SEM. Comparisons of continuous data were made using Kruskal–Wallis test or one-way ANOVA among three groups, and Mann–Whitney U test or unpaired t-test between two groups, where appropriate. The correlations between variables were determined by Spearman rank correlation coefficients. Categorical variables between two groups were examined using χ^2^ method and/or Fisher’s exact test, when appropriate. 

Multivariate logistic regression analyses were performed to determine factors independently associated with development of RPT > 10 mm. Variables of significance in the univariate analysis were entered into a binary logistic regression analysis. Results of multivariable analyses are reported as odds ratios (OR) with 95% confidence intervals and *p*-values. The optimal sensitivity, specificity and cutoff value of pleural fluid variables to predict RPT > 10 mm were evaluated with the receiver operating characteristics (ROC) by analyzing the area under the curve. A two-tailed *p*-value < 0.05 was considered to be statistically significant.

## 5. Conclusions

In conclusion, thrombin mediates dual upregulation of PAI-1 expression and MMT in human PMCs through PAR-1 signaling and may contribute to pleural fibrosis in TBPE. Inhibition of thrombin/PAR-1 axis may confer potential therapy for pleural fibrosis. More translational works and clinical studies are needed for validation.

## Figures and Tables

**Table 1:** Demographics, pleural fluid characteristics, and effusion levels of thrombin, fibrinolytic factors and cytokines among all patients (*n* = 46) ^†^.

	TPE	TBPE		*p*-Value *
		RPT ≤ 10 mm	RPT > 10 mm	
Subject, n	22	14	10	
Age, years	80 (65–88)	64 (20–87)	79 (34–91)	0.186
Males, n	14	9	6	0.830
Symptom onset to enrollment, days	7 (5–10)	14 (6–20)	16 (7–25)	0.304
Pleural fluid				
pH value	7.39 (7.30–7.46)	7.37 (7.25–7.46)	7.30 (7.23–7.37)	0.010
Glucose, mg/dL	141 (102–193)	126 (68–175)	88 (25–181)	0.105
Protein, g/L	2.3 (1.1–3.6)	4.7 (3.3–5.8)	4.6 (3.6–6.5)	0.829
LDH, IU/dL	83 (22–153)	274 (121–1370)	495 (215–2001)	0.064
Leukocyte count, cells/μL	313 (108–1080)	959 (235–5840)	1531 (137–6872)	0.667
ADA, IU/L	13 (6–36)	112 (59–262)	180 (68–220)	0.045
Thrombin, pg/mL	0.7 (0.1–1.1)	5.0 (3.9–5.4)	5.7 (5.4–6.7)	<0.0001
PAI-1, ng/mL	36.6 (3.1–140.5)	107.2 (40.4–150.1)	137.5 (100.9–225.3)	0.003
tPA, ng/mL	12.1 (6.9–35.0)	24.1 (1.0–56.9)	14.1 (1.8–41.5)	0.186
TNF-α, pg/mL	17.0 (4.5–109.8)	37.7 (15.0–184.4)	88.8 (35.8–287.2)	0.025
IL-1β, pg/mL	1.7 (1.4–3.7)	5.9 (4.3–34.3)	17.6 (5.8–63.1)	0.021
Initial pleural effusion CXR score, %	49 (25–65)	43 (29–59)	61 (42–93)	0.003
Residual pleural shadowing CXR score, %	0 (0–1)	2.0 (0.6–4.0)	4.5 (2.5–5.9)	<0.001
FVC at 12 months, % predicted	79 (77–82)	80 (78–81)	74 (71–76)	<0.001

Definition of abbreviations: TBPE, tuberculous pleural effusion; RPT, residual pleural thickening; LDH, lactate dehydrogenase; ADA, adenosine deaminase; PAI-1, plasminogen activator inhibitor-1; t-PA, tissue-type plasminogen activator; TNF-α, tumor necrosis factor-α; IL-1β, interleukin-1β; CXR, chest radiograph; FVC, forced vital capacity. ^†^ Data expressed as median (range). * Comparison between TBPE with RPT ≤ 10 mm and TBPE with RPT > 10 mm groups.

**Table 2:** Correlation between thrombin and inflammatory parameters, fibrinolytic factors and cytokines in TBPE (*n* = 24).

Variables	Coefficient *	*p*-Value
pH value	−0.46	0.051
Glucose, mg/dL	−0.16	0.460
LDH, IU/dL	0.35	0.070
ADA, IU/L	0.40	0.063
PAI-1, ng/mL	0.65	<0.0001
tPA, ng/mL	−0.04	0.862
TNF-α, pg/mL	0.38	0.066
IL-1β, pg/mL	0.41	0.058

Definition of abbreviations: TBPE, tuberculous pleural effusion; LDH, lactate dehydrogenase; ADA, adenosine deaminase; PAI-1, plasminogen activator inhibitor-1; tPA, tissue-type plasminogen activator; TNF-α, tumor necrosis factor-α; IL-1β, interleukin-1β. * Spearman correlation coefficient.

**Table 3:** Multivariate logistic regression analyses of factors associated with RPT > 10 mm among TBPE patients (*n* = 24).

Variables	Thrombin Excluded			PAI-1 Excluded		
	OR	95% CI	*p*-Value	OR	95% CI	*p*-Value
Thrombin, U/mL				9.05	3.09–28.42	0.007
pH value	0.92	0.83–1.02	0.135	0.25	0.01–1.181	0.623
ADA, IU/L	1.29	0.77–1.83	0.609	1.12	0.99–1.26	0.721
TNF-α, pg/mL	1.33	0.91–1.95	0.147	1.04	0.95–1.09	0.925
IL-1β, pg/mL	1.02	0.98–1.07	0.272	1.08	0.95–1.18	0.182
PAI-1, ng/mL	2.26	1.05–4.87	0.037			

Definition of abbreviations: RPT, residual pleural thickening; TBPE, tuberculous pleural effusion; OR; odds ratio; CI, confidence interval; ADA, adenosine deaminase; TNF-α, tumor necrosis factor-α; IL-1β, interleukin-1β; PAI-1, plasminogen activator inhibitor-1.

## Abbreviations

α-SMA	α-smooth muscle actin
CBB	Carbon black/bleomycin
ECM	Extracelluar matrix
MMT	Mesothelial–mesenchymal transition
MTBRa	*Mycobacterium tuberculosis* H37Ra
PAI-1	Plasminogen activator inhibitor-1
PAR-1	Protease-activated receptor-1
PMC	Pleural mesothelial cell
RPT	Residual pleural thickening
TBPE	Tuberculous pleural effusion
TF	Tissue factor
TPE	Transudative pleural effusion
